# PB.19. Distortion conspicuity and cancer detection: a comparison of cranio-caudal and mediolateral oblique mammographic projections

**DOI:** 10.1186/bcr3723

**Published:** 2014-11-03

**Authors:** S George, E Muscat, M Sinclair, S Heller

**Affiliations:** 1 St George's Healthcare NHS Trust, London, UK

## Introduction

Mammographic distortions are an indicator of breast malignancy. However, whether distortions and their associated cancers are more readily appreciable on cranio-caudal (CC) or mediolateral oblique (MLO) projection is unexplored. Our study sought to investigate this in view of its potential importance for cancer detection.

## Methods

**A total of **156 mammograms from the South West London Breast Screening Service were anonymised and randomly ordered. The series included 79 consecutive studies with confirmed distortion and 77 normal studies. Three blinded readers were asked to establish the presence of distortion on separately displayed CC and MLO views, and to score distortion conspicuity (1 to 5). Readers also reported the degree of confidence (1 to 5) that identified distortions represented malignancy.

## Results

Distortion detection sensitivity was 75% for the CC projection and 64% for the MLO projection (*P *< 0.005) and specificity was 78% for the CC projection and 84% for MLO (*P *< 0.05). Cancer detection sensitivity was 86% for the CC projection and 75% for the MLO projection (*P *= 0.12). Positive predictive values were 0.14 and 0.11 respectively. Cancer detection specificity was 55% for the CC projection and 65% for MLO (*P *< 0.05). Negative predictive values were 0.98 and 0.97 respectively. In cases of biopsy-proven malignancy, readers were more confident of the presence of cancer on the CC compared with the MLO view (3.05 ± 0.28 vs. 2.22 ± 0.28; *P *< 0.05).

## Conclusion

The CC projection was associated with significantly higher distortion detection and higher cancer detection rate than the MLO view. This view was also associated with greater confidence of identifying proven malignancy amongst readers.

